# Elemental Interactions and Local Structures in Liquid Sb-As and Sb-Al-As Alloys: Insights from Ab Initio Molecular Dynamics and Experimental Studies on As Aggregation and Diffusion Behaviors

**DOI:** 10.3390/ma18071633

**Published:** 2025-04-03

**Authors:** Zongbo Li, Yan Feng, Qiyue Wu, Yufeng Wen, Xiang Peng, Richu Wang

**Affiliations:** 1 School of Materials Science and Engineering, Central South University, Changsha 410083, China; 2 National Key Laboratory of Science and Technology on High-Strength Structural Materials, Central South University, Changsha 410083, China; 3 School of Science, Kaili University, Kaili 556011, China

**Keywords:** AIMD, liquid Sb-based alloys, zone refining, elemental interactions, local structures

## Abstract

The local structure, element interactions, and electronic structure properties in Sb-As and Sb-Al-As melts were studied using ab initio molecular dynamics (AIMD) simulations. Sb-0.1wt%Al alloy was prepared using vacuum melting, and both pure Sb and Sb-0.1wt%Al alloys were subjected to zone refining experiments to investigate the effect of Al addition on the removal efficiency of impurity As. The results show that in the Sb-Al-As ternary melt, the interaction between Al and As atoms is stronger than the interactions between other solvent atoms. The introduction of Al disrupts the Sb-As and As-As bonds, promoting the formation of Al-As bonds, which alters the state of As atoms in the melt and subsequently affects their diffusion properties. The study elucidates the kinetic process of Al-As bond formation in the melt. The bond-angle distribution function and the coordination polyhedron sequence indicate that with the addition of Al atoms, the geometric configuration around As atoms in the Sb melt and the types and numbers of clusters undergo significant changes. A strong hybridization occurs between the 4p orbitals of As atoms and the 3p orbitals of Al atoms. Moreover, the noticeable charge accumulation between Al and As atoms suggests a strong interaction between them. The addition of aluminum increased the removal rate of arsenic impurities in antimony from 67.27% to 83.24%, significantly enhancing the efficiency of arsenic removal.

## 1. Introduction

High-purity antimony (Sb) plays a crucial role in modern high-tech industries, with a wide range of applications and significant technological impact [1,2]. Sb with a purity of 4 N (99.99%) is a key material in the manufacture of thermoelectric coolers and thermoelectric generators, particularly in Bi_2_Te_3_-based alloys, where it effectively enhances thermoelectric conversion efficiency, supporting energy-saving and environmentally friendly technologies [3]. Sb of even higher purity, 5 N, holds an irreplaceable position in the semiconductor industry [4]. As a critical component of compounds such as AlSb, InSb, and GaSb, it is used in the production of high-performance infrared detectors, diodes, and hall effect measurement devices, which play a central role in communication, sensing, and measurement technologies [5].

Currently, in the pyrometallurgical process, the As content in crude antimony (about 90% purity) is generally between 0.2% and 0.3% [6]. Although the hydrometallurgical process can yield Sb with 98.5% purity, the As content still ranges from 0.02% to 1% [7]. Even when the purity is increased to 99.8%, the As content in Sb (approximately 0.08%) remains relatively high. This indicates that regardless of the smelting process used, the removal of As remains a significant challenge. In the production of high-purity Sb, zone refining is ultimately employed to obtain a high-purity product [8]. The distribution coefficient of each impurity plays a decisive role in the final refining efficiency. However, As is difficult to remove during zone refining due to its effective distribution coefficient being close to 1 (0.8 ± 0.1) [6,9]. To achieve high-purity Sb, the As content must first be reduced to an acceptable low level before zone refining. Common methods for this include distilling SbCl_3_ or directly vacuum distilling Sb, both of which exploit the significant difference in vapor pressure between SbCl_3_ and other chlorides, as well as between Sb and other impurity elements [10]. Vacuum distillation is an efficient, simple, and energy-efficient separation technique, ideal for producing high-purity antimony. Under vacuum conditions, the volatility differences between antimony and impurities are fully utilized, allowing for the rapid production of high-purity antimony. However, a challenge remains, as the evaporation characteristics of Sb and As are very similar, making it difficult to effectively separate them [11]. Electrolytic refining [12] uses electrochemical principles to remove impurities such as Cu, Pb, and Fe from crude antimony, producing high-purity antimony. Although this method is effective, it has high energy consumption and expensive equipment costs, and improper handling of the electrolyte and anode slime may lead to environmental pollution.

According to previous studies [13], methods to suppress As evaporation and achieve its separation include the use of multi-stage condensation systems (through precise design and temperature control), or the addition of inhibitors (such as Al), which suppress As evaporation by forming non-volatile intermetallic compounds [14,15]. Among these, the addition of metallic elements is a promising approach due to its simplicity and low cost. Chen et al. [16] investigated the use of V as an additive in the Al-Si solvent refining process to improve the removal rate of B in Si, and found that the addition of V formed VB_2_ compounds, significantly enhancing the removal efficiency of B. However, there is currently a lack of atomic-scale studies to explore the mechanism by which additives influence the purification process. Therefore, future research should combine experimental and theoretical calculations to reveal the atomic-level mechanisms of additives, providing theoretical support for the development of efficient separation technologies.

In addition, according to thermodynamic calculations [17], when the interaction between two solute elements in the melt is stronger than their interaction with the solvent elements, the activity coefficients of the solute elements decrease, leading to a reduction in the equilibrium distribution coefficient. Conversely, the opposite is also true. This indicates that the interaction between solute elements alters the activity coefficients, thereby affecting their chemical potential and ultimately influencing the equilibrium distribution coefficient. Kagawa Akio et al. [18,19] demonstrated through thermodynamic calculations and experimental methods that the distribution coefficients of alloying elements in steel change with variations in carbon content. Determining the interaction between solute elements typically relies on extensive experiments, where the interaction coefficients are calculated by plotting the relationship between solute element activity and concentration. However, this method is prone to experimental errors and is cumbersome in terms of data acquisition. In contrast, the AIMD method [20], which does not rely on potential functions, can accurately simulate the interactions between solute elements in the melt. Louis J. Santodonato et al. [21] used the AIMD method to calculate the strong interactions between components in the AlCuCrCoFeNi high-entropy alloy system and successfully predicted the possible phase compositions that could form in the alloy.

In this study, AIMD simulations were employed to systematically investigate element interactions and local structures in Sb-As and Sb-Al-As melts. The study provides detailed insights into the interaction mechanism between Al and As atoms, as well as the influence of Al addition on the local structure and dynamics of As atoms. To validate the theoretical results, further zone-refining experiments were conducted by adding a small amount of high-purity Al to Sb, confirming that Al addition significantly enhances the removal rate of As. This research provides both theoretical and experimental support for the efficient separation of Sb and As.

## 2. Computational and Experimental Methods

### 2.1. Computational Details

Density functional theory [22] (DFT)-based AIMD simulations were carried out using the vienna ab initio simulation package (VASP-version 5.4.4) [23]. The projector augmented-wave (PAW) [24] method was employed to represent the core electrons, and the perdew-burke-ernzerhof (PBE) [25] exchange-correlation functional within the generalized gradient approximation (GGA) [26] was used to describe electron exchange and correlation effects. A plane-wave cutoff energy of 320 eV [27,28,29] was applied. The temperature was controlled using a classical NVT ensemble coupled to a Nosé-Hoover thermostat [30,31]. The equations of motion were integrated using the Verlet algorithm [32,33] with a time step of 3 fs.

**Figure 1:**
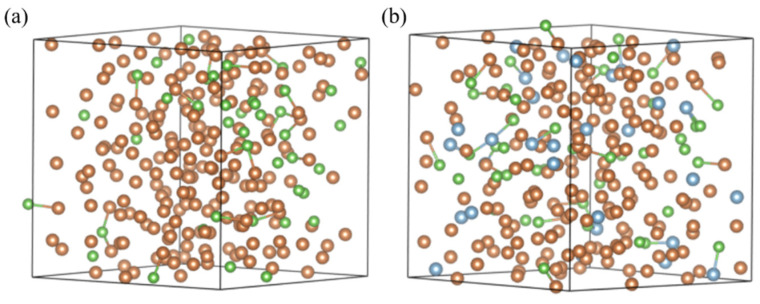
The supercell atomic configuration diagrams of Sb-As (**a**) and Sb-X-As (**b**) alloy melts at a temperature of 2000 K. Sb is represented in yellow, Al in blue, and As in green.

In the simulation study of the Sb-As and Sb-Al-As systems, we constructed a cubic supercell containing 256 atoms to analyze the structural evolution, focusing on the strong interaction mechanisms and their regulation of the melt’s microstructure. The study was carried out in two stages: In the initial stage, we established an Sb-As binary system model containing 40 As atoms for basic simulations. Building on this, the second stage involved the random substitution of 30 Sb atoms with Al atoms to create a ternary system, which was then further calculated. Figure 1 visually presents the initial configuration of the Sb-As binary and Sb-Al-As ternary supercells, with the atomic substitution strategy using the monte carlo method for random spatial distribution [34]. The initial configuration of the supercell was fully melted at 2000 K, followed by cooling at a rate of 1 × 10^7^ K/s down to 1600 K, 1500 K, 1400 K, and 1300 K. At each temperature, we adjusted the cell volume to bring the external pressure of the system close to zero (approximately ±200 MPa) [35]. Subsequently, the supercell underwent 10,000 steps of molecular dynamics (MD) simulation at each temperature to reach equilibrium. The last 5000 MD steps were selected to extract atomic trajectories, from which statistical analysis of the local structure was performed.

**Figure 2:**
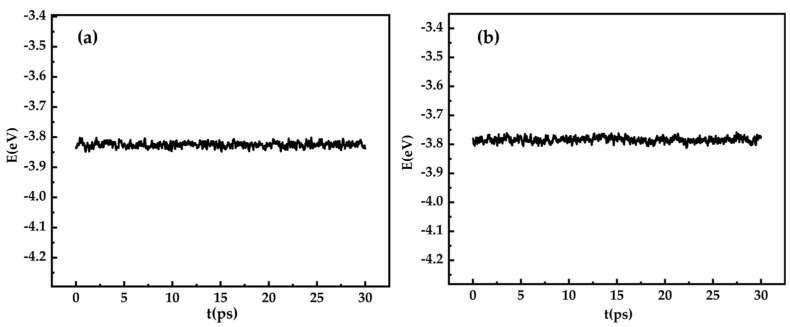
The time evolution of the total energy per atom for the liquid Sb_216_As_40_ (**a**) and Sb_186_As_40_Al_30_ (**b**).

The side lengths of the cubic supercells and the density of the melts for each system were determined using the “zero external pressure method”, Ref. [36] with detailed results presented in Table 1. The simulation was run for a total of 30 ps to ensure sufficient structural equilibration. Additionally, the comparison between the calculated pair distribution function g_Sb-As_ and the experimental [37] pair distribution function g_Sb_ of pure Sb suggests that the system likely achieved true ergodicity rather than being trapped in a metastable state. Figure 2 illustrates the variation in the total energy of each atom over time, with the amplitude of the total energy of the system controlled within 0.1‰. According to the general requirements for energy convergence testing in ab initio molecular dynamics simulations, the energy amplitude of the system should be less than 0.1‰, indicating that the system has reached thermal equilibrium. To investigate the interaction mechanisms between solutes in the Sb melt, the electronic density of states (DOS) was calculated [38,39]. For the Sb-Al-As system, a 5 × 5 × 5 Monkhorst-Pack k-point grid [40] was used to sample the Brillouin zone, with a cutoff energy set at 350 eV. From the last 200 configurations of the AIMD simulation, 20 configurations were randomly selected for DOS calculations. The electronic density of states in the melt was obtained by averaging the DOS of these 20 configurations.

### 2.2. Experimental Methods

The materials used in the experiment include commercial 3N5 pure antimony and an Sb-0.1wt%Al alloy prepared by mixing pure antimony with 5 N high-purity aluminum. The initial state of the materials was in the form of irregularly sized blocks. To ensure uniformity, the pure antimony was first melted. The total mass of the materials was 3.2 kg. The process began with vacuum pumping, and when the pressure reached 10 Pa, argon gas at 9000–10,000 Pa was introduced into the quartz tube to prevent the evaporation of antimony in the vacuum environment. The heating system was then activated to start the temperature rise, with the melting temperature set at 750 °C and a holding time of 1.5 h. During the melting process, the pressure was automatically controlled by the pressure control device. After the melting process was completed, the temperature was allowed to cool to room temperature before removing the pure antimony from the crucible. Subsequently, 999 g of the melted pure antimony and 1 g of high-purity aluminum were further melted for 1 h under the same conditions.

**Figure 3:**
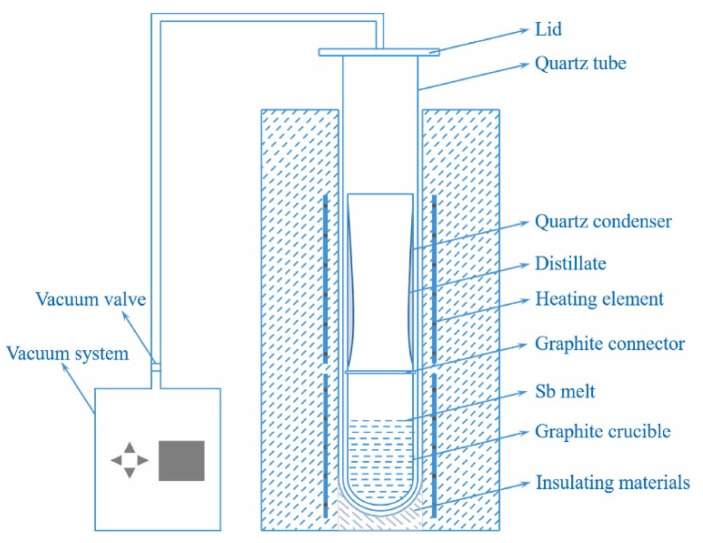
A detailed schematic diagram of the vacuum distillation furnace, illustrating the internal configuration with clarity.

**Figure 4:**
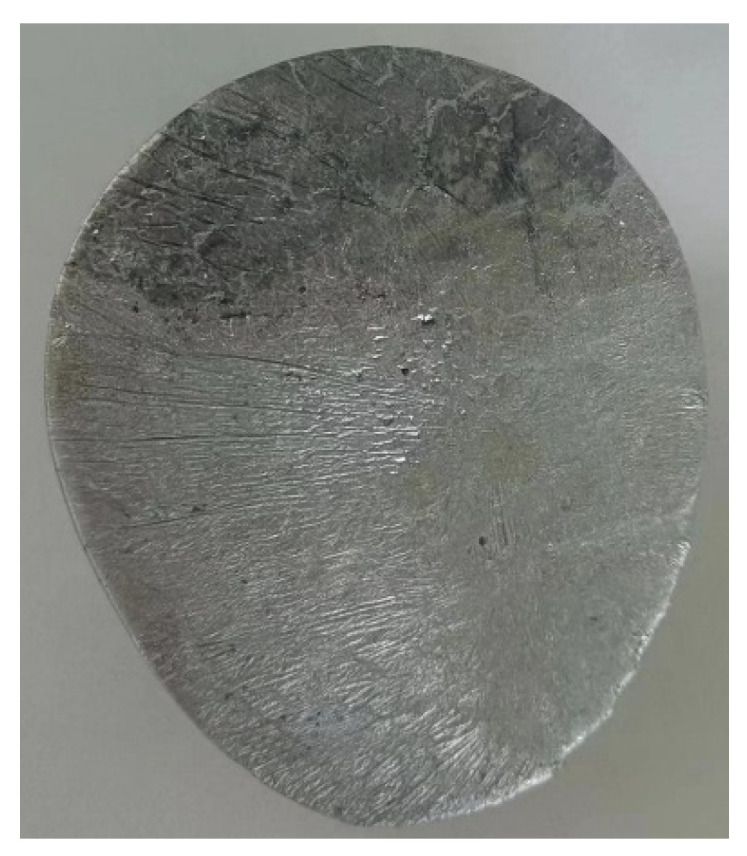
The prepared Sb-0.1wt%Al alloy ingot.

Figure 3 shows a schematic diagram of the self-assembled vacuum melting equipment, which consists primarily of a pressure control device, an argon gas tank, a vacuum system, a temperature control system, a high-purity graphite crucible, and insulating materials. The impurity content in the materials was determined using ICP-OES (SPECTRO Analytical Instruments GmbH, Kleve, Germany). Figure 4 shows the prepared Sb-0.1wt%Al alloy.

## 3. Results and Discussion

### 3.1. Pair-Correlation Function

**Figure 5:**
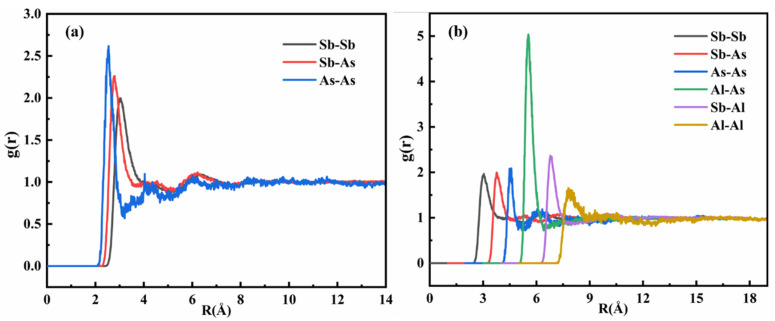
The partial binary correlation function of Sb-As (**a**) and Sb-Al-As (**b**) alloy melts; (**b**) the values of different systems are shifted by 1 unit along the *X*-axis.

Figure 5 presents the pair distribution functions [41] (PDFs) for the Sb-As and Sb-Al-As melts. In Figure 5a, the analysis of the Sb-As binary melt reveals three primary types of atomic interactions: Sb-Sb, Sb-As, and As-As. The intensity of the first peak in the pair distribution function serves as a quantitative indicator for assessing the relative strengths of interactions among solvent atoms, solute atoms, and solvent–solute pairs in the melt.

A detailed analysis of the pair distribution function for the Sb-As melt shows that the intensity of the first peak of g_As-As(r)_ is significantly higher than that of g_Sb-As(r)_ and g_Sb-Sb(r)_. This indicates that, in the molten state, the interaction strength between As-As atomic pairs is markedly stronger than that between Sb-As and Sb-Sb pairs. Furthermore, the intensity of the first peak of g_Sb-As(r)_ is slightly higher than that of g_Sb-Sb(r)_, suggesting that the interaction strength between Sb-As pairs is also somewhat greater than that between Sb-Sb pairs. In summary, the analysis of the pair distribution function for the Sb-As melt indicates that the solute As atoms exhibit a strong tendency for self-interaction, while their interactions with Sb atoms are relatively weak. This phenomenon suggests that, in the melt, As atoms are more inclined to form As-As bonds through self-interaction and aggregate into cluster structures, rather than forming Sb-As bonds with Sb atoms. These findings provide important insights for further understanding the microstructure and thermodynamic properties of the Sb-As melt.

Figure 5b shows the pair correlation function of the Sb-Al-As ternary melt, which clearly demonstrates that the local melt structure of As undergoes significant changes upon the addition of Al atoms. The intensity of the first peak in g_Al-As(r)_ is markedly higher than that of g_Sb-Sb(r)_, g_Sb-As(r)_, g_As-As(r)_, g_Sb-Al(r)_, and g_Al-Al(r)_, indicating that the interaction between Al and As atoms is stronger than the interactions between other elements in the Sb-Al-As ternary melt. As solute elements, the interaction between Al and As significantly exceeds their respective interactions with the solvent Sb atoms. The strong interaction between Al and As atoms leads to a significant change in the activity coefficient of As in the Sb matrix. This confirms that Al, as a solute element, plays a thermodynamically dominant role in the selective bonding with As. Based on thermodynamic analysis, the addition of Al will alter the equilibrium distribution coefficient of As in Sb. Moreover, due to the significant atomic interactions between Al and As in the Sb-Al-As ternary melt, the interaction between As and As atoms is weakened.

As shown in Figure 5b, the intensity of the first peak in g_As-As(r)_ is close to that of g_Sb-As(r)_ and g_Sb-Sb(r)_. Therefore, the stronger interaction between Al and As atoms causes a shift in As atoms that were originally inclined to bond with each other, to instead bond with Al atoms. The first peak of g_Al-As(r)_ is located at approximately 2.5 Å, and the first trough is located at about 3.4 Å. The position of the first peak represents the maximum possible bonding distance between the two atoms, i.e., the most likely distance. The position of the first trough represents the maximum interaction range in short-range order (the size of the nearest-neighbor ordering). Therefore, the most probable bonding distance between Al and As atoms is approximately 2.5 Å, indicating that the interaction range between Al and As is about 3.4 Å. This result indicates that the strong interaction between Al and As not only alters the local structure of the melt but also affects the distribution and bonding behavior of As atoms.

### 3.2. The Bonding Process of Al and As Atoms in an Sb Melt

**Figure 6:**
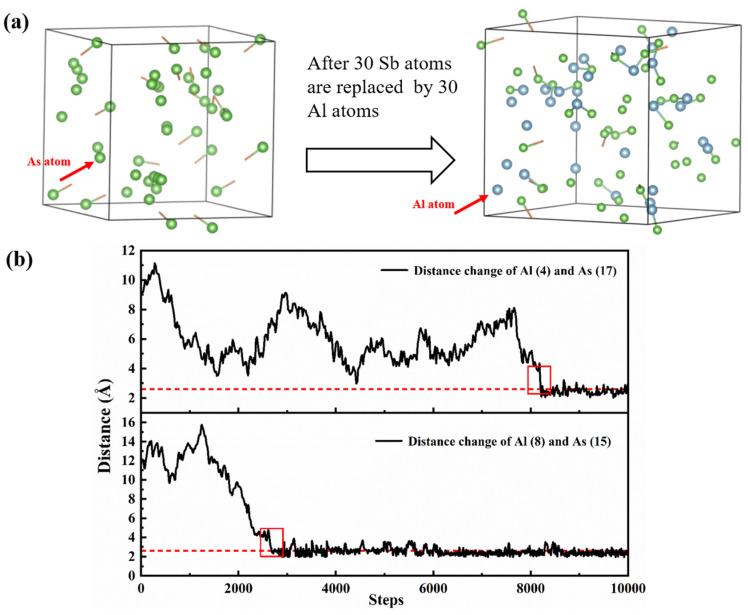
(**a**) Snapshots of the final step of the simulations for the Sb-As and Sb-Al-As systems. (**b**) Variation in the distance between Al and As atoms during the simulation of the Sb-Al-As system.

To provide a more intuitive description of the interactions between Al and As atoms in the aluminum melt, this study statistically analyzes the variation in the distance between Al and As atoms over time in the liquid phase and presents the related configurations in Figure 6. Figure 6a shows snapshots of the system before and after the addition of Al atoms. As seen in the figure, in the Sb-As system, when Al atoms are not present, As atoms exhibit a more clustered distribution in the melt, tending to form clusters. However, after the addition of Al atoms, the As-As atomic clusters are disrupted, and more As atoms bond with Al atoms, leading to a more uniform distribution of As atoms throughout the melt.

Figure 6b shows the variation in the distance between Al and As atoms throughout the entire simulation. For statistical analysis, we numbered 30 Al atoms and 40 As atoms in the system. As shown in Figure 6b, the distance between Al and As atoms eventually stabilizes at around 2.5 Å (indicated by the red dashed line). This distance corresponds to the position of the first peak in the pair correlation function. Figure 6b shows the variation in the distance between Al and As atoms throughout the entire simulation. For statistical analysis, we numbered 30 Al atoms and 40 As atoms in the system. As shown in Figure 6b, the distance between Al and As atoms eventually stabilizes at around 2.5 Å (indicated by the red dashed line). This distance corresponds to the position of the first peak in the pair correlation function g_Al-As(r)_ of the Sb-Al-As ternary melt, further confirming that the most likely bond length between Al and As is approximately 2.5 Å.

Additionally, Figure 6b shows that, during the early stages of the simulation, the distance between Al and As atoms is significantly greater than 2.5 Å, indicating that the Al and As atoms are initially farther apart in the starting configuration. Over the course of the simulation, they gradually move closer and aggregate. Notably, once the distance between Al and As atoms approaches 2.5 Å, it remains significantly stable throughout the subsequent simulations. This suggests that once Al and As atoms form a bond, it is difficult to break, further confirming the strong interaction between Al and As atoms of the Sb-Al-As ternary melt, further confirming that the most likely bond length between Al and As is approximately 2.5 Å. Figure 6b also shows that the distance between the Al and As atoms does not change gradually. Before the Al and As atoms form a chemical bond, their distance varies randomly. However, once the distance between the Al and As atoms becomes smaller than 3.4 Å, it rapidly decreases to 2.5 Å (indicated by the red rectangle).

### 3.3. Honeycutt–Andersen Index Analysis

**Figure 7:**
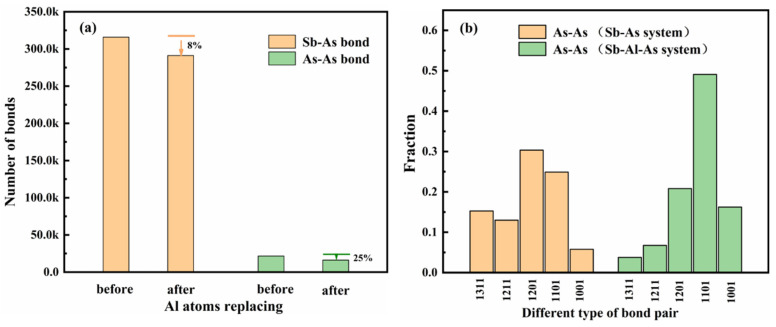
Shows the variation in the number of bond pairs (**a**) and the changes in bond pair types and contents (**b**) during the Sb-As and Sb-Al-As melt simulations.

**Figure 8:**
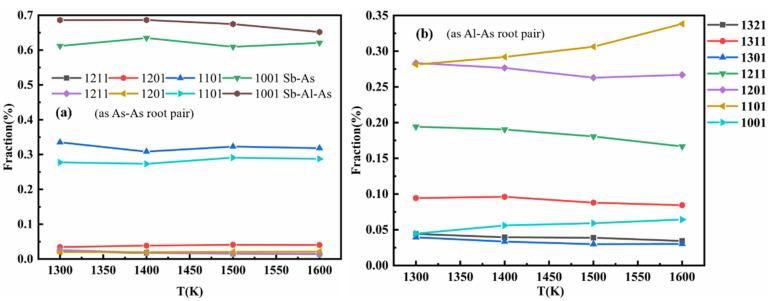
The change in the proportion of different types of bonding pairs (root bonds) in the Sb melt with temperature, such as (**a**) As-As and (**b**) Al-As, is as follows.

To clearly describe the effect of adding Al atoms on the local molten structure of As atoms, this study conducted a Honeycutt–Andersen [42] statistical analysis to investigate the changes in the number of Sb-As and As-As bonds before and after the addition of Al atoms, as well as the variation in bond types and quantities around the As atoms in the local structure with temperature. The analysis results are shown in Figure 7 and Figure 8.

Figure 7a shows the difference in the number of Sb-As and As-As bonds before and after the addition of Al atoms. Figure 7b analyzes the variation in the content of different types of bonding pairs, using As-As bonds as the root bonding pair. The results indicate that after the addition of Al atoms, the number of As-As bonds significantly decreases. This reduction is primarily due to the involvement of Al atoms, which leads to the rearrangement of some As atoms that were originally involved in As-As bonding. These As atoms form new Al-As bonds with Al atoms, partially replacing the original As-As and Sb-As bonds. As shown in Figure 7b, using As-As bonds as the root bonding pair, the introduction of Al atoms causes significant changes in both the number and types of bonding pairs. When As-As bonds are used as the root bonding pair, after adding Al atoms, the number of high-index bonding pairs (such as 1311 and 12,111) decreases significantly, while the number of low-index bonding pairs (such as 1101 and 1001) increases markedly, particularly with a significant rise in the 1101 bonding pair. In summary, these results demonstrate that the addition of Al atoms significantly disrupts the cluster structure formed by As atoms, providing strong evidence for the strong interaction between Al and As.

As shown in Figure 8a, after the addition of Al atoms, the content of low-index bonding pair 1001 significantly increases when using As-As bonds as the root pair, while the content of high-index bonding pairs 1101 and 1201 decreases significantly. Furthermore, in the Sb-Al-As ternary melt, the types of bonding pairs formed with Al-As as the root bond differ significantly from those formed with As-As as the root bond. From Figure 8b, when using Al-As as the root bond, the content of low-index bonding pairs (such as 1001, 1101, and 1201) decreases noticeably, while the content of the 1211 bonding pair increases significantly, with high-index bonding pairs such as 13XX even emerging. This suggests that, compared to As-As bonds, the local structure around Al-As bonds is more ordered, and the clusters are more tightly bound. Overall, these results reflect the significant impact of Al atoms on the local structure in the Sb-Al-As system, indicating that Al atoms not only participate in bond rearrangement but also promote the formation of a more ordered and stable local structure by altering the distribution of bonding pairs.

### 3.4. The Bond-Angle Distribution Function Analysis

**Figure 9:**
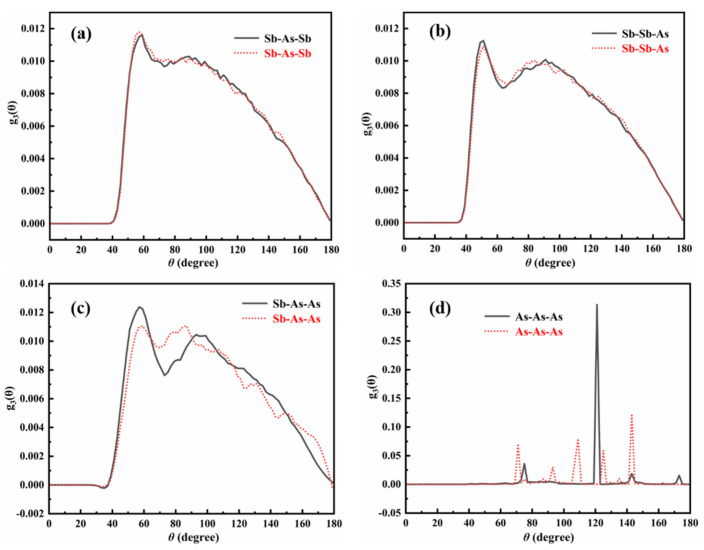
Effect of Al atoms on bond angle distribution in Sb-As melt: (**a**) Sb-As-Sb bond angle, (**b**) Sb-Sb-As bond angle, (**c**) Sb-As-As bond angle, (**d**) As-As-As bond angle.

In this study, the bond-angle distribution function (BADF) [43,44] was used to analyze the geometric configuration changes in the local coordination environment of As atoms in the Sb-As melt after the addition of Al atoms. The results are shown in Figure 9. Figure 9 presents the distribution of four bond angles associated with As atoms in the melt: Sb-As-Sb, Sb-Sb-As, Sb-As-As, and As-As-As. The black solid line represents the distribution of these four bond angles in the Sb-As binary melt, while the red dashed line represents the corresponding bond angle distribution in the Sb-Al-As ternary melt after the addition of Al atoms.

Figure 9a,b shows that the addition of Al atoms does not significantly affect the distribution of Sb-As-Sb and Sb-Sb-As bond angles. However, as illustrated in Figure 9c,d, the addition of Al atoms has a pronounced impact on the bond angle distribution of Sb-As-As and As-As-As, with the effect being particularly notable for As-As-As bond angles. Specifically, the introduction of Al atoms disrupts the As-As bonds, leading to substantial changes in the bond angle distribution involving As-As bonds. The intensity of small-angle Sb-As-As bond angles decreases significantly, while the intensity of large-angle As-As-As bond angles also shows a noticeable reduction. Although several higher peaks appear in the large-angle As-As-As bond angles, these peaks do not exhibit regularity because the bond-angle distribution function only reflects the probability of a specific bond angle occurring among all angles, rather than the absolute number of occurrences of that specific angle during statistical analysis. These results further indicate that Al atoms have a significant disruptive effect on As-As bonds. As Al atoms disrupt the As-As bonds, As atoms, which are more prone to forming clusters in the melt, gradually bond with Al atoms to form new bonding structures, thereby breaking the cluster structure among As atoms.

Overall, the disruptive effect of Al atoms on As-As bonds is highly significant. Al atoms not only break the As-As and Sb-As bonds but also promote the formation of new bonds between As and Al atoms, thereby disrupting the cluster structure that As atoms would typically tend to form in the melt.

### 3.5. Coordination Polyhedrons

**Figure 10:**
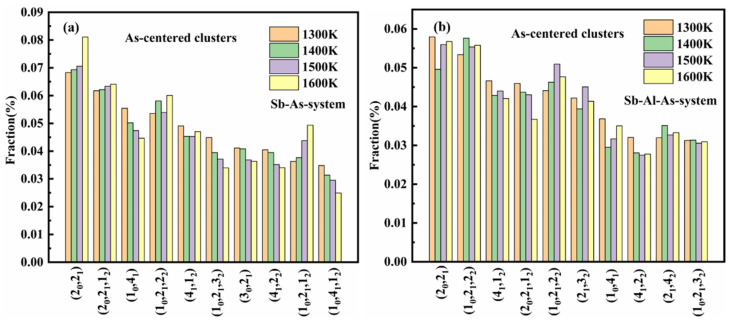
The fractions of coordination polyhedrons in Sb-As (**a**) melt and Sb-Al-As (**b**) melt at different temperatures.

The coordination polyhedral order in the melt was classified using the coordination polyhedron order characterization method defined in the literature [45]. The study shows that the Sb melt exhibits a rich diversity of coordination polyhedral order types, and more than 1000 different coordination polyhedral orders were identified at all the temperatures simulated in this study. Figure 10a,b displays the distribution of the top 10 most abundant coordination polyhedral order types at 1300 K under different temperatures. Although some of these polyhedral orders do not consistently occupy the most abundant position at different temperatures (1300–1600 K), they remain common types across all temperatures. The distribution characteristics of these coordination polyhedral orders effectively reflect the changes in the local geometric structure of the Sb melt at different temperatures.

The analysis results indicate that, in the Sb-As binary melt, the main cluster types centered around As atoms are (2_0_,2_1_), (2_0_,2_1_,1_2_), and (1_0_,4_1_). These clusters are also present in the Sb-Al-As ternary melt, but their concentrations decrease to varying extents. Additionally, with the addition of Al atoms, the content of clusters such as (2_1_,4_2_), (2_1_,3_2_), and (1_0_,2_1_,2_2_) significantly increases. This suggests that the addition of Al not only alters the types of clusters but also significantly affects their distribution, thereby changing the local structural environment around As atoms. These changes reveal the substantial impact of Al atoms on the local structure of As atoms in the melt, indicating that the introduction of Al effectively facilitates the reorganization of cluster structures and the adjustment of local configurations.

### 3.6. Dynamical Properties

**Figure 11:**
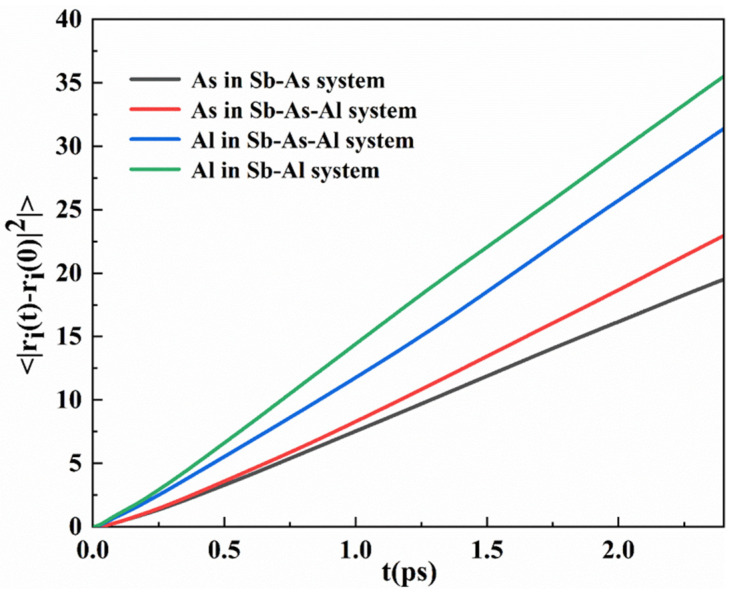
The MSD curves of Al and As atoms in the Sb-As, Sb-Al, and Sb-Al-As systems.

The interaction between Al atoms and As atoms in the melt inevitably affects their diffusion behavior. The diffusion rate of solute atoms in the melt is one of the key factors determining segregation efficiency. Therefore, this study focuses on the diffusion characteristics of As atoms in the melt. To calculate the diffusion coefficient of the solute atoms, the mean square displacement [46] (MSD) method was employed for analysis, and the MSD calculation results are shown in Figure 11.

The data from Figure 11 and Table 2 indicate that in the Sb-Al and Sb-As binary systems, due to the lack of Al-As interactions, the diffusion coefficient of Al atoms (2.565 Å^2^/ps) is significantly higher than that of As atoms (1.432 Å^2^/ps). However, in the Sb-Al-As ternary system, the strong Al-As interaction leads to a significant change in the diffusion behavior: the diffusion coefficient of Al atoms decreases to 2.211 Å^2^/ps, a reduction of approximately 14%, while the diffusion coefficient of As atoms increases to 1.662 Å^2^/ps, an increase of approximately 16%. The underlying mechanism behind this phenomenon can be further analyzed as follows: during the diffusion process, Al atoms must overcome the strong interactions with As atoms, which increase the energy barrier for diffusion, thereby reducing the diffusion rate of Al atoms. Specifically, the Al-As interaction may restrict the free movement of Al atoms by forming temporary coordination structures (such as short-range order), making their diffusion path more convoluted and requiring higher energy. In contrast, the diffusion of As atoms appears to benefit from the Al-As interaction. Al atoms may act as a “bridge” in the melt, reducing the energy required for the diffusion of As atoms by coordinating with them, thereby facilitating the smoother movement of As atoms. This synergistic effect may result from the combined influence of the high electronegativity of Al atoms and the electronic structure characteristics of As atoms.

### 3.7. Electronic Properties

To further investigate the microscopic mechanism of bonding between Al and As atoms, we conducted a systematic analysis of the partial electronic density of states in the Sb-Al-As ternary melt system after the addition of Al atoms.

**Figure 12:**
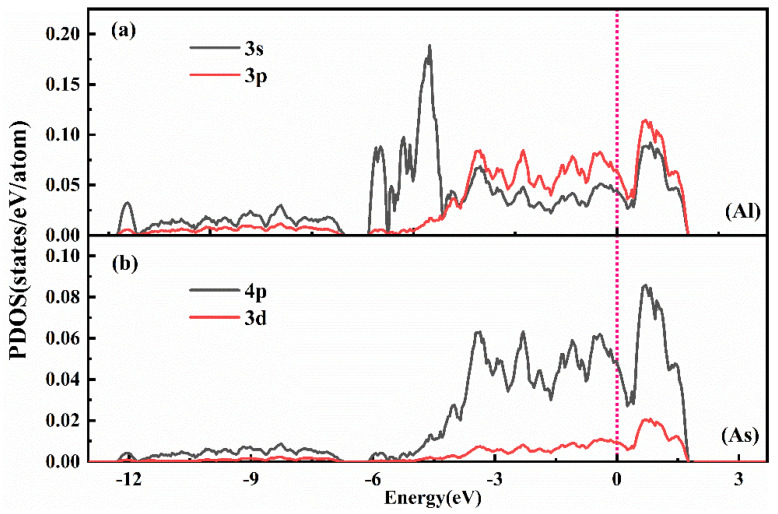
The partial densities of states (PDOS) of Al (**a**), As (**b**) atoms in Sb-Al-As melts. The vertical dashed lines indicate the zeroed Fermi level.

Figure 12 shows the partial electronic density of states (PDOS) of the Al-3p, Al-3s orbitals, as well as the As-4p and As-3d orbitals. In Figure 12a, several distinct overlapping peaks appear in the energy range from approximately 0 to −4 eV below the Fermi level for the Al-3s and Al-3p orbitals, indicating strong 3s(Al)-3p(Al) electronic hybridization within the Al atoms. This hybridization enhances the electronic activity of Al atoms, providing a basis for their interaction with As atoms. Further analysis reveals significant overlapping peaks in the PDOS of the Al-3p orbitals and As-4p orbitals in the energy range from approximately 0 to −4.5 eV below the Fermi level, suggesting 4p(As)-3p(Al) electronic hybridization between the As-4p orbitals and the Al-3p orbitals. This hybridization is the primary source of the strong interaction between Al and As, explaining the observed strong bonding between Al and As atoms in this system. Additionally, although the contribution of the As-3d orbitals near the Fermi level is relatively weak, it may still influence the Al-As interaction to some extent. Based on the above analysis, the strong interaction between Al and As atoms primarily arises from the 4p(As)-3p(Al) electronic hybridization, while the 3s(Al)-3p(Al) hybridization within the Al atom further strengthens this interaction. Through this analysis, we gain a clearer understanding of how Al and As atoms form strong chemical interactions via hybridization between their electronic orbitals, thereby stabilizing the structure of the ternary melt system.

**Figure 13:**
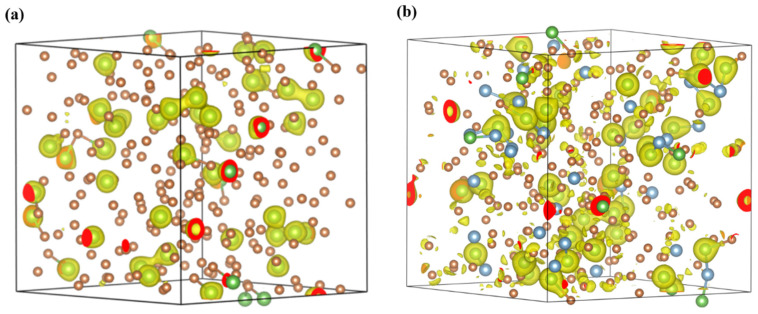
The charge densities of Sb-As (**a**) and Sb-Al-As (**b**) melts. Sb is represented in yellow, Al in blue, and As in green.

Figure 13a shows the charge density distribution [47] in the Sb-As melt, which clearly reveals the distribution of electron density and highlights the distinct covalent bonding characteristics between Sb and As atoms. This is primarily attributed to the p-p orbital hybridization between the 4p orbitals of As and the 5p orbitals of Sb, which enhances the bonding interaction and increases the charge energy between Sb and As atoms, exhibiting a certain degree of covalent interaction. Additionally, significant high-electron density regions are observed between As-As atoms, indicating charge localization between the As atoms and the formation of localized electron-rich regions. This localized charge distribution can be interpreted as a charge transfer phenomenon, further confirming the strong interaction between As atoms.

In contrast, the charge density distribution of the Sb-As-Al ternary melt shown in Figure 13b reveals a significant high electron density region around the Al-As atoms, indicating a stronger interaction between Al and As atoms. This phenomenon contrasts with the Sb-As melt, where the high electron density is primarily concentrated between the As-As atoms. Therefore, it can be inferred that the bonding strength of the Al-As bond is stronger than that of the As-As bond. This difference may arise from the introduction of Al atoms, which alters the electronic structure of the system, causing a redistribution of electron density, thereby enhancing the interaction between Al and As atoms while weakening the interaction between As atoms. In conclusion, by comparing the charge density distributions of the Sb-As binary melt and the Sb-As-Al ternary melt, we can conclude that the addition of Al significantly alters the electronic structure and atomic interactions of the system, strengthening the Al-As bond and relatively weakening the As-As bond. This provides important microscopic insights into the influence of Al on the properties of the Sb-As system.

### 3.8. Zone Melting Experiment and Result Analysis

**Figure 14:**
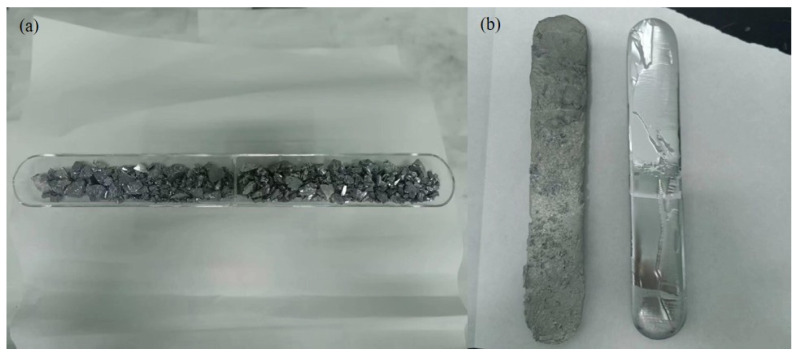
(**a**) Quartz boat containing raw materials. (**b**) Antimony ingots after zone refining. Left: Sb-0.1wt%Al, right: Sb.

The steps of the zone refining [48] experiment are as follows:

(1) First, wipe the quartz tube and quartz boat twice with anhydrous ethanol, then place the quartz boat containing the raw material at the appropriate position in the quartz tube. One end of the quartz tube is connected to the hydrogen gas inlet, while the other end is sealed with a flange that has an outlet.
(2) Introduce hydrogen gas into the quartz tube and maintain it for 1 h, then begin heating. Once the temperature of the molten zone reaches the set value, start maintaining the temperature. After half an hour, begin the zone refining process. The state of the molten zone is observed every half hour, and the temperature of the molten zone is adjusted as needed. The speed of the first pass is 75 mm/h with a molten zone width of 30–40 mm; the speed of the second pass is 30 mm/h with a molten zone width of 20–30 mm. And the duration of the process was approximately 120 h. After each pass, the heater returns to the starting point to continue the next pass. To improve the refining efficiency, each quartz tube is equipped with two heaters.
(3) After completing the two passes, turn off the heating and stop introducing hydrogen gas once the system has cooled down. Then, remove the ingot from the quartz boat, wipe it twice with anhydrous ethanol, and take a sample for analysis. The sampling position is at the center of the ingot, and ICP-OES is used to detect the impurity content. The results are shown in Table 3, and the antimony ingot after zone refining is shown in Figure 14b.

Table 3 presents the average As content and standard deviation of the Sb and Sb-0.1wt%Al samples before and after regional refining, based on the results of three independent experiments. For the Sb sample, the arsenic content decreased from 33.00 ± 1.73 ppm before refining to 10.80 ± 1.11 ppm after refining, with an arsenic removal efficiency of approximately 67.27%. For the Sb-0.1wt%Al sample, the arsenic content decreased from 34.00 ± 3.61 ppm before refining to 5.72 ± 0.45 ppm after refining, with a removal efficiency of approximately 83.24%. These results indicate that the regional refining process effectively reduced arsenic content in multiple trials, with minimal variability in the data, thereby validating the reliability of the arsenic removal efficiency.

On the other hand, the introduction of Al altered the surface morphology of pure antimony. As shown in Figure 14b, the surface of pure Sb after zone refining is smooth and mirror-like, while the surface of the Sb-Al alloy after zone refining appears wrinkled and uneven. This is likely due to the formation of Sb-Al compounds (such as AlSb) during the zone refining process. The density of these compounds (4.18 g/cm^3^) is lower than that of antimony (6.18 g/cm^3^), causing them to float to the surface of the melt during the refining process, which results in a surface that differs from the smooth surface observed after refining pure Sb.

## 4. Conclusions

This study employs the AIMD method to investigate the local structure, element interactions, and electronic structure properties of Sb-As and Sb-Al-As melts. Sb-0.1wt%Al alloy was prepared via vacuum melting, and regional melting experiments were conducted on both pure Sb and Sb-0.1wt%Al alloy to explore the impact of Al addition on the impurity As removal efficiency.

Through the study of the pair correlation functions and the bonding process between Al and As atoms in Sb-As and Sb-Al-As melts, it is found that in the ternary melt, there exists a significant interaction between Al and As atoms, with their interaction strength surpassing that of Sb-Al, Sb-As, and As-As atomic interactions. The introduction of aluminum disrupts the As-As and Sb-As bond structures, promoting the formation of Al-As bonds. This study reveals the dynamics of Al and As atom bonding, clearly demonstrating the dynamic changes in their interaction and the characteristic distance between Al and As atoms. The BADF indicates that the introduction of aluminum significantly alters the geometric configuration around As atoms in the Sb melt. These changes affect the thermodynamic properties of As atoms, alter their state of existence in the melt, and subsequently influence their diffusion characteristics, which in turn impacts the equilibrium distribution coefficient of As. The coordination polyhedron sequence further reveals that with the addition of Al, the types and quantities of clusters surrounding As atoms in the Sb melt undergo significant changes.

Electronic structure analysis reveals that the 4p orbitals of As atoms hybridize with the 3p orbitals of Al atoms, forming strong electronic orbital coupling. A significant accumulation of high-density charge is observed around the Al and As atoms, further confirming the strong interaction between Al and As atoms. Moreover, after zone refining treatment, the As content in pure Sb decreased from 33.00 ppm to 10.80 ppm, with a removal efficiency of 67.27%; in the Sb-Al alloy, the As content decreased from 34.00 ppm to 5.72 ppm, with a removal efficiency of 83.24%. These results demonstrate that the addition of Al significantly enhances the efficiency of As removal from Sb.

## Figures and Tables

**Table 1:** The supercell edge length, density, and pressure in Sb-based melts at different temperatures.

Systems	Temperature (K)	Length (Å)	Density (g/cm^3^)	Pressure (MPa)
Sb_186_As_40_Al_30_	1600	20.39	5.18	54
	1500	20.30	5.25	181
	1400	20.21	5.32	192
	1300	20.13	5.39	−143
Sb_210_As_40_	1600	20.53	5.62	21
	1500	20.38	5.75	103
	1400	20.28	5.83	74
	1300	20.17	5.93	197

**Table 2:** The diffusion coefficient (Å^2^/ps) of B and Ti atoms in different systems.

Method	Atoms	Sb-Al System	Sb-As System	Sb-Al-As System
MSD	Al	2.565	/	2.211
	As	/	1.432	1.662

**Table 3:** As content (ppm) before and after zone refining. Values represent the average of three independent trials ± standard deviation.

	Sb	Sb-0.1wt%Al
As before zone refining	33.00 ± 1.73	34.00 ± 3.61
As after zone refining	10.80 ± 1.11	5.72 ± 0.45

## Data Availability

The original contributions presented in this study are included in the article. Further inquiries can be directed to the corresponding authors.
